# Asking the better question - analytic approaches to failed ventilation transitions

**DOI:** 10.1186/s13054-026-06442-7

**Published:** 2026-11-12

**Authors:** Carmen A. T. Reep, Harm-Jan de Grooth, Diederik Gommers, Leo Heunks, Evert-Jan Wils

**Affiliations:** 1 Department of Intensive Care, Erasmus Medical Center, Rotterdam, The Netherlands; 2 Intensive Care Center, UMC Utrecht, Utrecht, The Netherlands; 3 Department of Intensive Care, Radboud University Medical Center, Nijmegen, The Netherlands; 4 Department of Intensive Care, Franciscus Gasthuis & Vlietland, Rotterdam, The Netherlands

**Keywords:** Mechanical ventilation, Controlled mode, Ventilator weaning, Airway extubation, Switch failure

Current literature is dominated by analyses of failed transitions from controlled to assisted ventilation, consistently linking failure to worse outcomes, including higher mortality rates [1–6]. These findings could be interpreted as supporting a more cautious switching strategy to prevent such failures. We argue, however, that this inference is not supported by the underlying analytical approaches, for reasons deeper than the usual association-is-not-causation critique. In this commentary, we explain the limited clinical relevance of current approaches, propose more informative analytic strategies to address concerns about switch failures, and illustrate their application in exploratory analyses.

Throughout our discussion, *switch failure* refers to the return to controlled ventilation following a switch attempt, under the assumption that this return is triggered either because deterioration occurred after the switch or because clinicians judged that the physiologic response to the switch was likely to become harmful. Although patients may occasionally return to controlled ventilation for other reasons, switch-failure research is primarily concerned with post-switch clinical deterioration, and this interpretation is adopted here.

## Critical reflection on current failed switch studies

Available studies linking failed switches to worse outcomes all compare failed switches to successful ones [1–6]. While such comparisons are not inherently problematic, they are not clinically informative. Imagine two patients who appear equally ready to switch from controlled to assisted ventilation. After switching, one deteriorates and is returned to controlled ventilation, while the other tolerates assisted ventilation. Unsurprisingly, the patient who failed has worse outcomes. This is, in essence, what success‑versus‑failure studies demonstrate: they show that switch failure is a marker of poor prognosis.

This comparison, however, does not tell us whether the switch attempt *itself* caused the poorer outcomes for the patient who failed. The deteriorating patient was already more vulnerable in ways detectable or undetectable before the switch. Switching merely reveals this pre-existing vulnerability, separating vulnerable patients from non-vulnerable patients by failure status. The success-versus-failure comparison therefore only shows that there is an association between vulnerability and poor outcome, not whether this outcome is caused by the vulnerability, the switch attempt, or a combination. Although it remains possible that the switch attempt itself may contribute to worse outcomes, comparing more vulnerable patients to less vulnerable patients simply cannot demonstrate this.

Even after perfectly matching patients who failed with those who succeeded, they still differ in some unmeasured way, otherwise one could not fail and the other succeed. This is why the problem with comparing patients based on their response to an exposure (success vs. fail) is not simply confounding: the comparison itself requires that some difference remains. Without such remaining differences, matched patients would have the same outcome, and a success-versus-failure comparison would not exist.

Importantly, even established failure-associated harm does not justify a more cautious switching strategy, although it is often interpreted that way. Such reasoning overlooks the potential benefit of early switching for the patient who would succeed, a benefit that may outweigh the possible downside of a failure.

Together, these limitations render success‑versus‑failure comparisons of limited value for addressing the ongoing uncertainty surrounding the more informative bedside decision of *when to switch*.

## The more meaningful questions

A more informative descriptive contrast to approximate whether the switch *attempt* for patients who failed contributed to their poorer outcomes is to compare *switch attempts that failed* with *delayed switch attempts*. It allows us to ask whether, in hindsight, delaying would have been better for those who ultimately failed, directly addressing whether concerns of failure were valid. However, although this comparison now includes a control group of patients who did not undergo an early switch attempt, it remains associational rather than causal because it still conditions on the subgroup of patients who fail.

An even more meaningful analysis reflects the actual choice a clinician faces at bedside: *switch now* versus *delay switching*, irrespective of the eventual outcome of the switch. This contrast directly addresses whether the benefit of an early switch strategy for patients who would tolerate assisted ventilation outweighs the potential downside for those who would fail. We now have a relevant control group, and, by not conditioning on the future event of failure, the undetected pre-existing vulnerabilities discussed earlier do not lead to bias.

To address concerns about failure specifically, this comparison can be restricted to patients at highest risk of failing if switched early, identified solely from baseline (pre-switch) characteristics. Within this high-risk stratum, the net clinical benefit of early switching is expected to be smallest, as the absolute number of failures will likely be highest. This best approximates whether early switching remains advantageous in the subgroup where failure risk is highest.

Although failure currently cannot be predicted with high accuracy [1], it is still partially predictable, allowing us to identify patients with a higher probability of failure.

## Exploratory analyses addressing the more meaningful questions

Once the more meaningful questions are identified, observational data can be used to approximate an answer. We used the MIMIC-IV database [7], building on recent work suggesting benefit from earlier switching among patients ventilated for at least two days with a PaO_2_/FiO_2_ ratio > 150 mmHg [8]. Because the observed benefit of early switching may largely reflect outcomes among successful switch attempts, we examined whether this benefit also appears among early switchers who failed. For this, we applied the proposed comparisons outlined above.

We first analysed whether *switch attempts that failed* had worse outcomes compared to *delayed attempts*. After adjustment for baseline propensity to switch and predicted failure risk, no clear association with fewer ventilator-free days was observed (details in the Supplement).

Second, within this same population, we identified patients at higher risk of failure and compared a strategy of *early* versus *delay switching*. Within this subgroup, early switching was not associated with delayed successful extubation nor an increase in ICU mortality risk (details in the Supplement).

Taken together, both proposed approaches offer more clinically informative ways to address concerns about harm from failure than the failure‑versus‑success comparisons that currently dominate the literature. Our exploratory findings suggest that, within the eligible population we studied (PaO_2_/FiO_2_ >150 mmHg), attempting the switch is plausibly not associated with worse outcomes. Even when conditioning on failure, which would typically bias results toward worse outcomes, we did not observe clear evidence of fewer ventilator-free days among early switchers who failed, and in the highest-risk quartile we likewise saw no clear evidence of adverse outcomes among patients switched early. One possible explanation is that patients who deteriorate can be promptly returned to controlled ventilation, thereby limiting adverse consequences. These results should be interpreted as highly exploratory and were intended to guide more informative ways to examine harm from failure. Confidence intervals are wide, and non‑significant estimates should not be taken as evidence of no association.

As noted earlier, it remains possible that for patients with certain unobserved vulnerabilities, the switch attempt itself may contribute to worse outcomes. But because these vulnerabilities are unobserved, they are likewise not identifiable in practice at the moment a decision has to be made. Therefore, the best-we-can-do approach we discussed is also the most informative one: to evaluate treatment strategies using observable and measurable characteristics, and to assess whether the net benefit of trying and succeeding outweighs the potential downside of failing.

## Implications for future research

Future work should move away from success-versus-failure comparisons, which are of limited clinical relevance. Instead, studies should focus on the actual bedside decision: whether to attempt a switch despite the possibility of failure or to delay it. This is the question clinicians truly face, and the one most likely to generate clinically meaningful evidence and guidance.

## Supplementary Information

Below is the link to the electronic supplementary material.

Supplementary Material 1

## Data Availability

The data that support the findings of this study are available from PhysioNet (MIMIC-IV v3.1, https://physionet.org/content/mimiciv/3.1/).

## Abbreviations

ICU: Intensive care unit
