# Supplementary material for: Asking the better question - analytic approaches to failed ventilation transitions
Source: Crit Care. 2026 Nov 12;30:588. doi: 10.1186/s13054-026-06442-7 (PMC13757172; doi:10.1186/s13054-026-06442-7)
Supplement: Supplementary file 1 [file 13054_2026_6442_MOESM1_ESM.docx]

**Asking the Better Question - A Comment on Current Analytic Approaches to Failed Transitions to Assisted Ventilation**

*Carmen A T Reep; Harm-Jan de Grooth; Diederik Gommers; Leo Heunks; Evert-Jan Wils*

Supplemental materials

Contents

[Switch and fail versus delay	3](#_Toc243225633)

[Switch now versus delay in patients with a higher risk of failure	3](#_Toc243225634)

[Figures	5](#_Toc243225635)

[Tables	7](#_Toc243225636)

[References	8](#_Toc243225637)

# Switch and fail versus delay

We selected patients who had received ≥48 hours of controlled ventilation and had a PaO_2_/FiO_2_ ratio >150 mmHg. To explore whether patients who switched and subsequently failed had worse outcomes than those in whom switching was delayed, we compared early switchers who later failed with patients who did not switch within the early-switch window (irrespective of their later switching status). Switches within 24 hours of eligibility were classified as *early* (=*switch now*), with a 12-hour definition used as a sensitivity analysis.

To improve comparability between groups, we estimated two propensity scores based on baseline characteristics measured at eligibility: (1) the probability of undergoing an early switch and (2) among early switchers, the probability of subsequently failing. These probabilities were derived from respiratory, hemodynamic, and clinical variables: time since intubation, PCO_2_, pH, PaO_2_/FiO_2_ ratio, PEEP, driving pressure, respiratory rate, tidal volume, cardiovascular SOFA score, and sedation level (awake, moderate, or deep sedation). We then compared ventilator-free days (VFDs) using multivariable Poisson regression adjusted for both propensity scores. VFDs at day 28 were defined conventionally: patients who died within 28 days received 0 VFDs, while survivors were assigned the number of days alive and free from invasive mechanical ventilation within the first 28 days. Patients who remained ventilated for ≥28 days also received 0 VFDs. For this exploratory analysis only, we excluded patients lost to follow-up before 28 days without a recorded successful extubation, as ventilator-free days could not be reliably determined. Additionally, we excluded patients who died within the 24 (or 12 hour) period (the *grace period*) before having the opportunity to undergo the assigned switching strategy.

Uncertainty was quantified using cluster bootstrap resampling (150 iterations), with the entire analytical pipeline, including missingness imputation using MICE (m=1) and propensity score estimation, repeated within each bootstrap sample.

Results are as follows. Among 1,465 patients meeting eligibility criteria, we did not find a clear difference in ventilator‑free days between early switchers (≤24h) who subsequently failed compared with delayed switchers (rate ratio 0.97, 95% CI 0.52-1.60). Although the point estimate corresponds to a 3% lower expected VFD count, the wide confidence interval does not exclude clinically relevant benefit or harm. Findings were similar using the ≤12-hour definition (rate ratio 0.94; 95% CI: 0.66 to 1.63). These exploratory, non-causal analyses showed no clear association between attempting the switch and subsequently failing and worse outcomes. However, because the analysis conditions on a post-treatment variable (failure status), it cannot support causal conclusions.

# Switch now versus delay in patients with a higher risk of failure

Again, we selected patients who had received ≥48 hours of controlled ventilation and had a PaO_2_/FiO_2_ ratio >150 mmHg. The moment of meeting these eligibility criteria is referred to as ‘time zero’.

To avoid conditioning on post-treatment information (failure status), we approximated the effect of switch failure by comparing *switch now* versus *delay switching* among patients at higher risk of failure if switched at time zero. To identify this subgroup, we first fitted a penalized logistic regression model among early switchers to predict switch failure using variables known at time zero: respiratory, hemodynamic, and demographic variables: PaO_2_/FiO_2_ ratio, ventilatory ratio, tidal volume, pH, compliance, respiratory rate setting, PEEP, days since intubation, sedation, age, sex, BMI, asthma, COPD, CHF, and cardiovascular SOFA score. These were chosen because they capture the respiratory mechanics, gas‑exchange status, and baseline patient characteristics most relevant for discriminating early failure risk. Predicted failure probabilities were then calculated for all eligible patients at time zero, and those in the highest quartile (Q4) were classified as high risk. Because these analyses were exploratory, we did not perform external validation, which would be required before using such a model to identify truly high‑risk patients in practice.

Within this high-risk subgroup, we compared *early* (=*switch now*) versus *delayed* switching using a target trial emulation based on the clone-censor-weight approach[1]. Analyses were adjusted for the following factors: time since intubation, PCO_2_, pH, PaO_2_/FiO_2_ ratio, PEEP, driving pressure, respiratory rate, tidal volume, cardiovascular SOFA score, and sedation level (awake, moderate, or deep sedation), selected in consultation with clinicians because these pre‑treatment variables reflect the physiologic and clinical factors that influence both the decision to switch and subsequent outcomes.

We classified switches within 24 hours of eligibility as early, with a 12-hour threshold used in a sensitivity analysis. Outcomes were 28-day ICU mortality risk and the Restricted Mean Time Lost (RMTL) to successful extubation, which reflects the average time successfully extubated during the 28-day follow-up. Higher RMTL values indicate earlier or more frequent successful extubation.

For successful extubation, death was treated as a competing event; for ICU mortality, ICU discharge was treated as a competing event because it indicates survival. Uncertainty was quantified using cluster bootstrap resampling (150 iterations) across the entire analysis pipeline, with missing values imputed using MICE (m = 1), and prediction model development and high-risk subgroup selection repeated within each bootstrap sample.

Results are as follows. Although switch failure was not highly predictable, discrimination was better than chance (ROC-AUC 0.65, 95% CI 0.61-0.69). Patients in the highest-risk quartile (Q4) had a failure rate of 65%, compared with 58% in the overall population. They also switched later, with a median time from eligibility to switch of 43 hours (IQR 19-85) versus 23 hours (IQR 9-55) in the overall population. Compared with the overall population, Q4 patients had slightly lower PaO_2_/FiO_2_ ratios, higher PEEP, respiratory rates, cardiovascular SOFA scores, and sedation levels, and were more likely to have COPD, sepsis, emergency surgery, and ECMO support (Table 1).

In the high-risk subgroup (n=366), the RMTL of successful extubation was 16 days (95% CI 11-19) for early switchers (≤24h; n=116) versus 14 days (95% CI 12-16) for delayed switchers (n=261), corresponding to a mean difference of 2 days over 28 days (95% CI -2 to 5; P=0.12) (Figure 1, Table 2). ICU mortality risk at day 28 was 16% (95% CI 7-27) for early switchers and 15% (95% CI 8-23) for delayed switchers, yielding an inconclusive difference of 1% (95% CI -11 to 17; P=0.96) (Figure 2, Table 2). Covariate balance was achieved after adjustment (Figure 3). Results were similar using the ≤12-hour definition (Table 2).

Although exploratory, these findings suggest that, even among patients at highest risk of switch failure, earlier switching is plausibly not associated with delayed successful extubation or increased ICU mortality compared with delaying the switch.

# Figures

**Figure 1**. **Probability of successful extubation among patients at high risk of switch failure**. Weighted cumulative incidence curves comparing early (≤24h) versus delayed (>24h) switching among patients in the highest quartile of predicted failure risk who met eligibility criteria (PaO_2_/FiO_2_ >150 mmHg, provided two days invasively mechanically ventilated). Early switching was associated with a non-significant 2-day increase in restricted mean time to successful extubation over 28 days (95% CI: -2 to 5 days; P=0.12).

**Figure 2. Mortality risk among patients at high risk of switch failure**. Weighted cumulative incidence curves comparing early (≤24h) versus delayed (>24h) switching among patients in the highest quartile of predicted failure risk who met eligibility criteria (PaO_2_/FiO_2_ >150 mmHg, provided two days invasively mechanically ventilated). Early and delayed switching were associated with similar 28-day ICU mortality, with an estimated risk difference of 1% (95% CI, -11% to 17%; P=0.96).

**Figure 3. Covariate balance plot**. Standardized mean differences comparing early (≤24 h) versus delayed (>24 h) switching strategies before (unadjusted) and after (adjusted) covariate adjustment at day 1 following eligibility, when most switches occurred. Standardized mean differences between −0.1 and 0.1 (dashed vertical lines) were considered indicative of adequate covariate balance.

# Tables

|  | Full patient population | Q4 highest probability of failure |
| --- | --- | --- |
|  | (N=1465) | (N=366) |
| Sex female, No. (%) | 587 (40.1%) | 140 (38.3%) |
| Age, years, median (IQR) | 61.0 (48.0, 72.0) | 54.0 (40.0, 65.0) |
| Body-mass index, kg/m^2^, median (IQR) | 28.4 (24.6, 34.6) | 33.4 (27.2, 40.7) |
| Hours since intubation, median (IQR) | 48.0 (48.0, 51.0) | 48.0 (48.0, 60.0) |
| Presence of tracheostomy, No. (%) | 0 (0%) | 0 (0%) |
| Received ECMO, No. (%) | 40 (2.7%) | 25 (6.8%) |
| Cause of ICU admission, n (%) |  |  |
| Hypercapnic Respiratory Failure | 119 (8.1%) | 35 (9.6%) |
| Hypoxaemic Respiratory Failure | 419 (28.6%) | 124 (33.9%) |
| Respiratory Failure (Hypercapnic or Hypoxaemic) | 547 (37.3%) | 135 (36.9%) |
| Sepsis/septic shock | 617 (42.1%) | 186 (50.8%) |
| Cardiogenic pulmonary edema | 313 (21.4%) | 76 (20.8%) |
| Cardiac arrest | 194 (13.2%) | 54 (14.8%) |
| Emergency surgery | 144 (9.8%) | 45 (12.3%) |
| Elective surgery | 31 (2.1%) | 9 (2.5%) |
| Shock (other than septic) | 455 (31.1%) | 123 (33.6%) |
| Trauma | 483 (33.0%) | 117 (32.0%) |
| Respiratory parameters, median (IQR) |  |  |
| Set respiratory rate, breaths/min | 20.0 (16.0, 24.0) | 24.0 (20.0, 28.0) |
| FiO_2_ | 40.0 (40.0, 50.0) | 50.0 (40.0, 60.0) |
| PaO_2_:FiO_2_ ratio, mmHg | 221 (184, 273) | 201 (170, 243) |
| PEEP, cmH_2_O | 8.00 (5.00, 12.0) | 14.0 (12.0, 16.0) |
| Driving pressure, cmH_2_O | 11.5 (10.0, 14.0) | 11.0 (9.00, 13.0) |
| Tidal volume, mL/kg of PBW | 6.73 (6.07, 7.80) | 6.47 (5.95, 7.32) |
| Compliance, mL/cmH_2_O | 35.9 (28.3, 47.4) | 33.8 (28.2, 47.3) |
| Ventilatory ratio | 1.53 (1.23, 1.89) | 1.77 (1.45, 2.25) |
| Blood gas, median (IQR) |  |  |
| SaO_2_, % | 97.0 (96.0, 98.0) | 97.0 (95.0, 98.0) |
| PaO_2_, mmHg | 113 (94.0, 149) | 98.5 (84.3, 128) |
| PaCO_2_, mmHg | 40.0 (35.0, 45.0) | 42.0 (37.0, 48.0) |
| pH | 7.39 (7.34, 7.43) | 7.37 (7.33, 7.42) |
| Coexisting illness, n (%) |  |  |
| COPD | 142 (9.7%) | 46 (12.6%) |
| Asthma | 162 (11.1%) | 45 (12.3%) |
| Heart failure: NYHA classes III-IV | 451 (30.8%) | 107 (29.2%) |
| Cardiovascular SOFA score, No. (%) |  |  |
| <1 | 378 (25.8%) | 47 (12.8%) |
| 1-3 | 172 (11.7%) | 37 (10.1%) |
| >3 | 508 (34.7%) | 221 (60.4%) |
| Sedation level, No. (%) |  |  |
| Awake | 144 (9.8%) | 24 (6.6%) |
| Moderate sedation | 645 (44.0%) | 147 (40.2%) |
| Deep sedation | 529 (36.1%) | 169 (46.2%) |

***Table 1*: Baseline characteristics.** Patient characteristics at the time patients first met switch eligibility criteria, shown for the overall study population and for patients in the highest quartile (Q4) of predicted switch failure risk.

| **Switch eligibility** | **Definition early** | **Successful extubation RMTL difference, days (95% CI); P** | **ICU mortality risk difference, % (95% CI); P** |
| --- | --- | --- | --- |
| PaO_2_/FiO_2_ >150 mmHg | ≤24h | 2 (-2 – 5); 0.12 | 1 (-11 – 17); 0.96 |
|  | ≤12h | 1 (-3 – 4); 0.4 | 1 (-10 – 18); 0.88 |

***Table 2*:** **Outcomes in patients at Q4 high risk of switch failure**. Comparison of early (=*switch now*) versus delayed switching in the population 2 days intubated with a PaO_2_/FiO_2_ > 150 mmHg who were in the highest quartile of predicted switch failure risk. Weight-adjusted outcome differences were estimated over 28 days of follow-up. Positive differences in the Restricted Mean Time Lost (RMTL) of successful extubation indicate earlier or more frequent successful extubation with early switching, whereas positive ICU mortality risk differences indicate higher mortality with early switching. CI = confidence interval; P = p-value.

# References

[1]	Reep CAT, Wils E-J, Fleuren LM, Breskin A, Bellani G, Laffey JG, et al. Early vs. Delayed Switching from Controlled to Assisted Ventilation: A Target Trial Emulation. Am J Respir Crit Care Med 2025. https://doi.org/10.1164/RCCM.202406-1162OC.
